# Scoping review: Machine learning interventions in the management of healthcare systems

**DOI:** 10.1177/20552076221144095

**Published:** 2024-10-22

**Authors:** Oritsetimeyin V Arueyingho, Anmar Al-Taie, Claire McCallum

**Affiliations:** 1 School of Computer Science, Electrical and Electronic Engineering, and Engineering Maths (SCEEM), Centre for Doctoral Training in Digital Health and Care, University of Bristol, UK; 2 Department of Clinical Pharmacy, Faculty of Pharmacy, Istinye University, Istanbul, Turkey

**Keywords:** Machine learning, healthcare management, big data, digital health

## Abstract

**Background:**

Healthcare institutions focus on improving the quality of life for end-users, with key performance indicators like access to essential medicines reflecting the effectiveness of management. Effective healthcare management involves planning, organizing, and controlling institutions built on human resources, data systems, service delivery, access to medicines, finance, and leadership. According to the World Health Organization, these elements must be balanced for an optimal healthcare system. Big data generated from healthcare institutions, including health records and genomic data, is crucial for smart staffing, decision-making, risk management, and patient engagement. Properly organizing and analysing this data is essential, and machine learning, a sub-field of artificial intelligence, can optimize these processes, leading to better overall healthcare management.

**Objectives:**

This review examines the major applications of machine learning in healthcare management, the algorithms frequently used in data analysis, their limitations, and the evidence-based benefits of machine learning in healthcare.

**Methods:**

Following PRISMA guidelines, databases such as IEEE Xplore, ScienceDirect, ACM Digital Library, and SCOPUS were searched for eligible articles published between 2011 and 2021. Articles had to be in English, peer-reviewed, and include relevant keywords like healthcare, management, and machine learning.

**Results:**

Out of 51 relevant articles, 6 met the inclusion criteria. Identified algorithms include topic modelling, dynamic clustering, neural networks, decision trees, and ensemble classifiers, applied in areas such as electronic health records, chatbots, and multi-disease prediction.

**Conclusion:**

Machine learning supports healthcare management by aiding decision-making, processing big data, and providing insights for system improvements.

## Introduction and background

Healthcare systems have evolved differently in contrasting countries and their individual histories are often intertwined with community and societal structure development which are largely influenced by culture, religion, and politics.^1^ For example, the National Health Service (NHS) that was commissioned in 1948 was founded on the Beveridge report on Social Insurance and Allied Services^2^ and was influenced by the ‘liberal’ foundation of William Beveridge.^3^ The importance of good health and living conditions cannot be overemphasized, hence, while it has become a focus for politicians in their attempt to win over electorates, organisations such as the World Health Organisation (WHO) are continuously introducing guidelines and health promotion strategies to ensure that quality of lives improve. The WHO defined health systems to include organizations, resources, people, and institutions whose primary objectives are to improve health, either by direct health improvement activities or indirect influence on the determinants of health.^4^ However, while healthcare systems might have different behaviours the WHO has established that they should all have similar building blocks. These building blocks include health workforce, service delivery, health financing, access to essential medicines, health information systems, and leadership/governance.^4^ Healthcare management is a universal term that refers to the coordination of these building blocks to achieve specific outcomes, it goes beyond leadership/governance. Planning, organizing, staffing, directing, and controlling which are key elements of management are prerequisites for optimum healthcare management.

Regardless of the suggestion and implementation of several health management strategies e.g., healthcare management through organizational stimulation,^5^ there is still no perfect healthcare system. This is because systems vary in complexity.^6^ Also, there have been no conclusive findings on what health system is better or worse in different countries after extensive research, and even countries with similar systems vary in levels of quality^7^ . Out-of-pocket payments are on the rampage in underdeveloped and developing countries like Nigeria and Cameroon,^8^ high cost of care and poor insurance coverage are still challenges in the United States,^9^ and staff shortages and coping with an ageing population are challenges in the United Kingdom.^10^

 Figure 1

**Figure 1.**
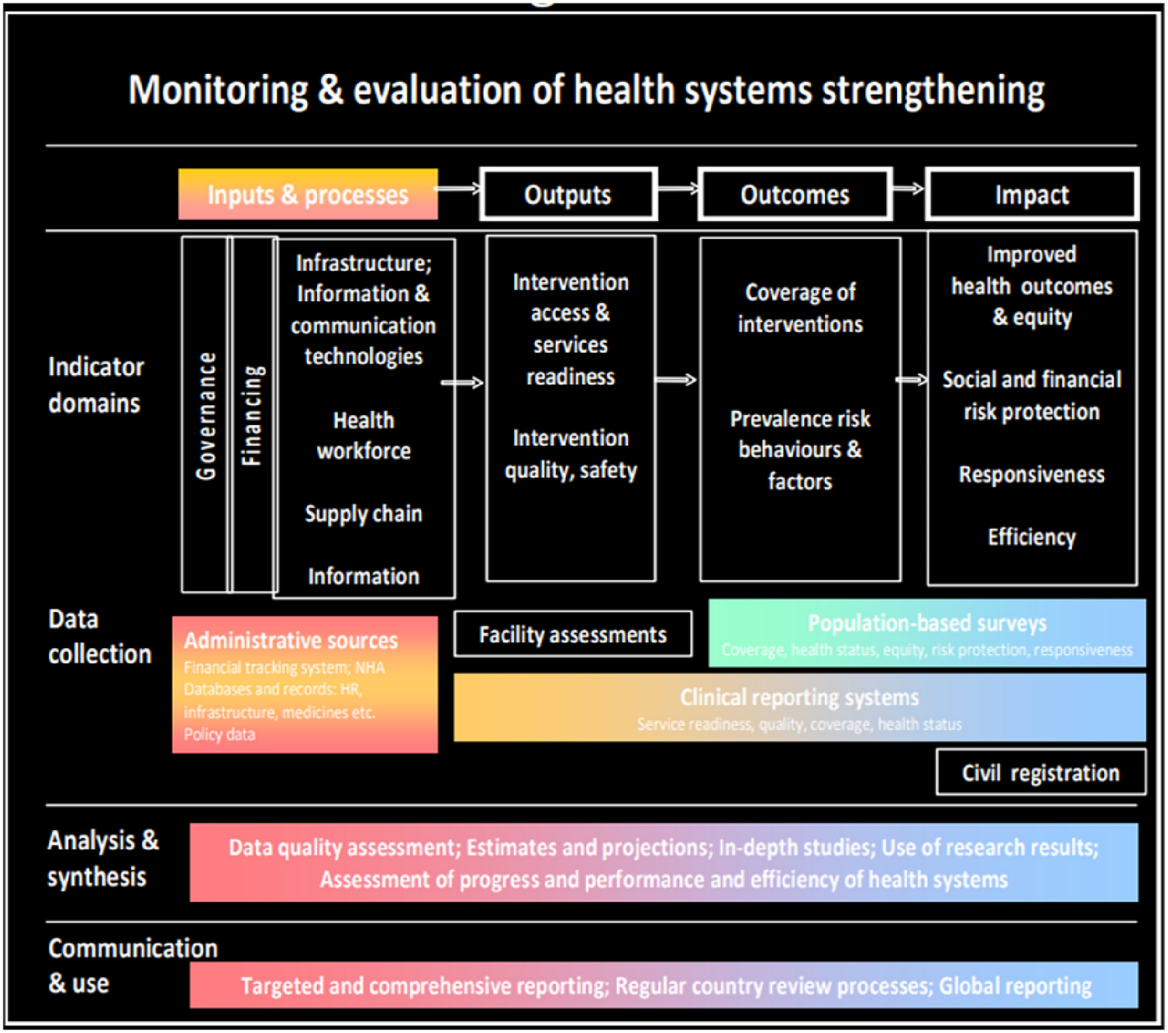
Monitoring and evaluation of health systems strengthening. Source:.^11^.

The figure above explicitly describes how a healthcare system can be strengthened. Inputs and processes which should be overseen by a solid managerial framework in the aspects of health workforce, infrastructure and other necessary building blocks would result in desired outcomes such as guaranteed access to healthcare and increased safety. These outcomes could be further assessed using data collection. To evaluate their impact levels, further analysis and synthesis of extracted data could also be used to make predictions, with the end results being apt communication to necessary stakeholders and comprehensive reporting. Facility assessments, clinical reporting systems and population-based surveys are some of the methods of data collection, depending on the tier of healthcare management being assessed. Regardless, large volumes of data would be generated to make global reports and influence policy creation. Due to the nature of data being generated from the various nodes of healthcare systems, it would be unrealistic to use traditional data processing methods to yield great results. Due to the complexity of managing healthcare systems, some researchers have made several suggestions one of which was the creation of an e-healthcare management system that would be based on service and cloud-oriented architecture.^12^

### Big data, machine learning and healthcare management

Big Data can be defined as any form of data that has these three basic characteristics: high velocity, large volume, and wide variety.^13^ Big Data is usually too large or complex to be processed using traditional techniques e.g., relational database management systems. Ideally, data could either be structured, unstructured or semi-structured. Structured data is characterized by a defined format and length, e.g., patient biodata forms that have specified spaces for names, date of birth, address, sensor data from Radio Frequency Identification (RFID) tags, etc..^14^ Research has proven that it accounts for only 20% of data, compared to unstructured data that makes up the other 80%. Unstructured data on the other hand neither has a defined format nor length, it includes images, videos, audio files, random posts, General Practitioner (G.P) notes, prescriptions etc. Semi-structured data falls in between these two broad categories and might include data that exist in the form of key-value pairs.

Big data is not a solitary form of technology however, it has evolved overtime to becoming an important necessity. These periods of evolution occurred in different ‘waves’, the first of which was the creation of manageable data structures as described in the relational model of data for large-shared data banks.^15^ Flaws in the relational model saw the introduction of the entity-relationship model,^16^ and when the volume of data expanded data warehouses were created. With time, unstructured data increased in capacity due to the growth of the internet and other web-based platforms, even within the health industry. This led to the migration of a more unified model, that encouraged data storage using metadata and exploration of other forms of web/internet-based storage systems.

The analysis of Big Data provides information into past, present, and future statistics that could be used to make better decisions, and usually it is classified into data analytics and data science both of which are interconnected.^17^ While data analytics focuses on present and past data collection and interpretation, data science involves the use of explorative analytics to predict the future.^18^ Reports that influenza outbreaks in the United States could be predicted by Google at least 7–10 days before the Centre for Disease Control (CDC) by monitoring phrases associated with common searches for the onset of influenza made a lot of people realize the potential of analysing large data sets.^19^

 Figure 2

**Figure 2.**
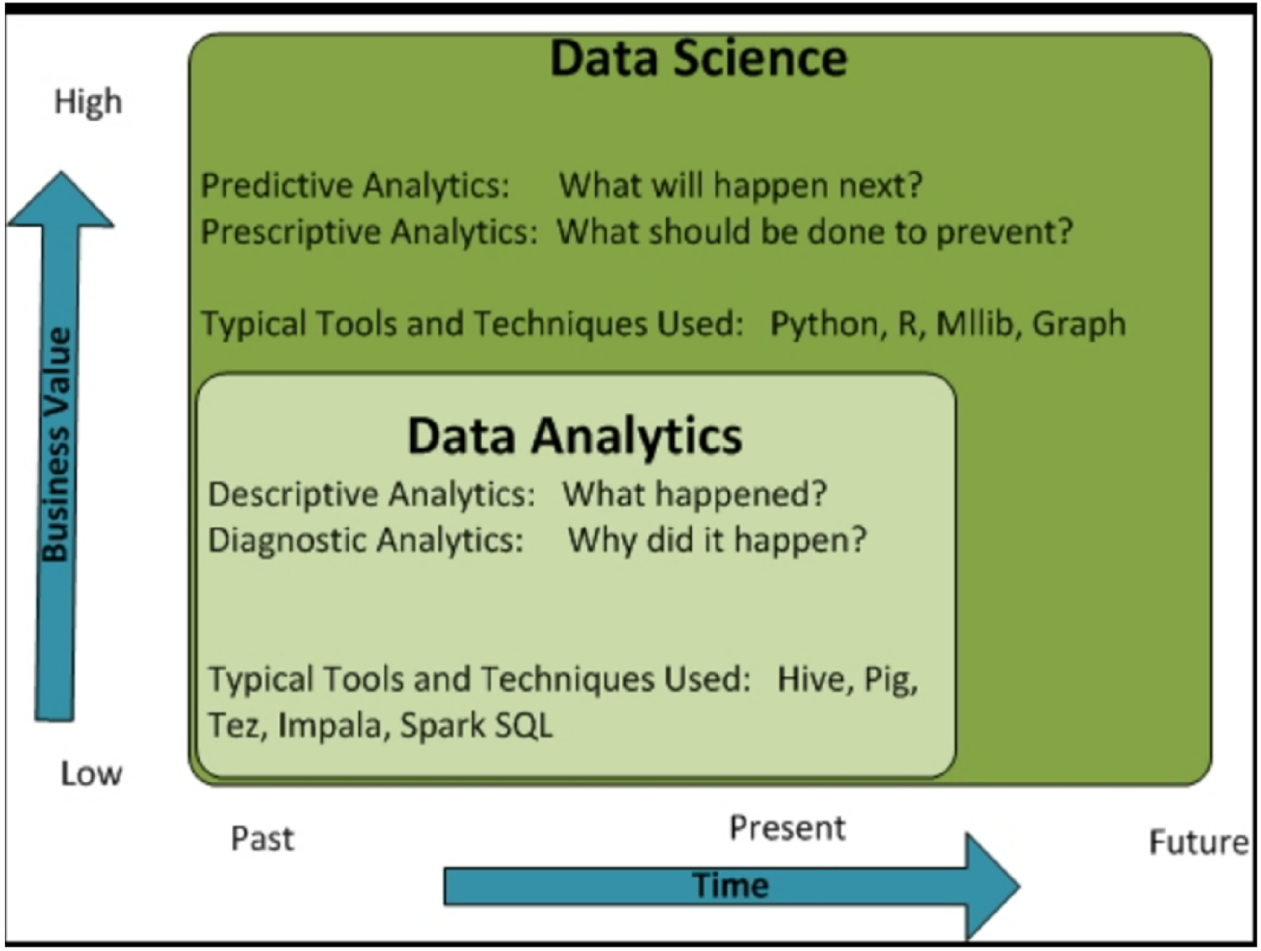
Comparison between data science and data analytics. Source:.^17^.

Arthur Samuels definition of machine learning (ML) is often quoted because of its simplicity, even though it aptly communicates the rudiments of this concept. He describes it as a field of study in which machines/computers are taught to learn without being programmed.^20^ Essentially, it involves the development of algorithms into which big data is fed for the purpose of solving a particular task. The machine/computer would utilize this data and ‘learn’ from it; hence, data could be seen as an experience which is directly proportional to its human-like efficiency.^21^ There are broadly four types of machine learning algorithm which include supervised machine learning, unsupervised, semi-supervised and reinforcement. Regression and Classification are the two fundamental types of supervised ML, they require ‘supervision’ as past labelled data is being ‘fed’ into the algorithm to make future predictions.^22^ Examples of algorithms that fall within this category include linear regression, logistic regression, support vector machines and a practical example of their implementation is predicting the acceptance of new digital health tools in a hospital based on the different fields or disciplines of staff.

Unsupervised ML involves the feeding of unlabelled data into an algorithm which tries to work on its own to discover patterns, clustering and dimensionality reduction are some major techniques used under this category.^23^ Semi-supervised ML sits between the supervised and unsupervised methods and tries to strike a balance between the two, e.g., by combining labelled and pseudo-labelled sets of data to generate predictive and descriptive results which are characteristics of the first two algorithm types. In Data science, regression and classification are of primary importance. Python is the tool that is often used in machine learning, and in the analysis of health-related data, several steps must be followed. These steps include the importation of relevant libraries used in data science, such as scikit learn, pandas, numpy and matplotlib, then the importation of the data set. The data set would be cleaned, arranged, and properly filtered using these tools before being divided into training and testing sets. Usually, 80% of the data is used to train the algorithm model and 20% is used for testing,^24^ subsequently predictions could be made and visualized using the libraries responsible for this function.

It has been established that healthcare systems generate tonnes of Big Data due to the automation of a lot of systems^25^ Data could be generated from public health systems, insurance companies, hospitals, research, patients themselves, laboratories, pharmacies, and even non-classical healthcare players. All these data could be collected, archived, analysed, and dutifully utilized. There is a plethora of applications of Big Data in Healthcare Management, most of which were unimaginable decades ago. The more health care data is being recorded; the more intentional decisions could be made. It is important to note that machine learning could be utilized in the creation of predictive models in healthcare systems, which would optimize and enhance the quality of every stage of the healthcare process.^26^ While we see great advancements made in data science, machine learning and the optimization of big data in a variety of fields including health institutions, it would be interesting to know if attention has been paid to managerial contexts, being consciously aware that a solid managerial framework could contribute to the success of an institution.^27^ There have been multiple articles on the use of machine learning in the different sub-fields of healthcare institutions and it is still important to note them, however, the core functions of a manager which include planning, organising, staffing, controlling and decision making could be influenced by the optimization of machine learning.^28–31^

 Figure 3

**Figure 3.**
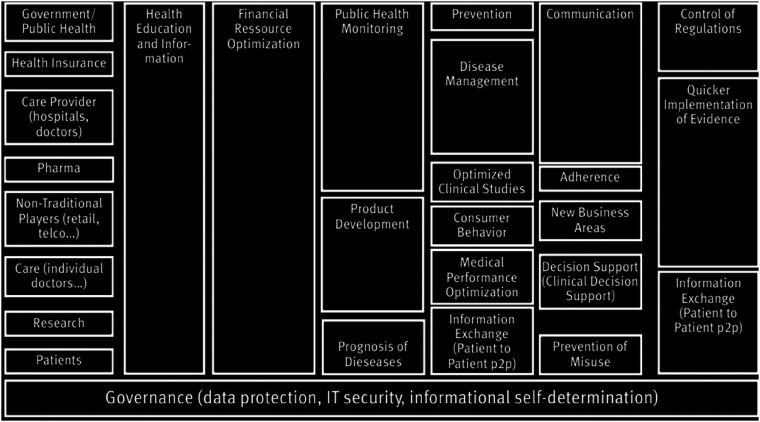
Fields of applications of big data in healthcare management. Source:.^28^.

***Objectives****.* This research article is interested in exploring the use of artificial intelligence in the form of machine learning to solve issues related to health data analysis, integration, and visualization in the management of healthcare systems, and the extent to which this could mitigate challenges associated with ensuring that the objectives of managing health systems are achieved. This article is also aimed at reviewing literature that provides information on the algorithms and/or other techniques used in the management of healthcare data, their limitations and supposed benefits.

## Methods

Guidelines prescribed in the PRISMA checklist were utilised in the conduction of this scoping literature review, and unlike meta-analyses there was no need to synthesize data. There was also no need for a quality assessment as the intention was to derive a collective comprehension of the efforts of other researchers to explore the impact of the use of machine learning in the management of healthcare systems.

The following activities were conducted for the purpose of generating this systematic review: Research questions, strategy for article search, selection criteria and abstraction of Data:

### Research questions

This systematic review is based on the following research questions that are in line with the research objectives:

1. What are the major applications of machine learning in healthcare management?
2. What algorithms/other methods are frequently used in the analysis of healthcare data?
3. What are the existing limitations of these algorithms and/or other machine learning interventions?
4. Based on evidence, what are the benefits of the utilization of machine learning in the management of healthcare systems

### Strategy for article search

Databases that were explored include: SCOPUS (Elsevier), IEEE Xplore, SpringerLink, and ScienceDirect. Keywords used in the search include ‘healthcare management’, ‘health management’, ‘machine learning’, and ‘big data’. Search strings used include: ‘healthcare management and machine learning’, ‘machine learning, big data in healthcare systems’, and ‘management of healthcare systems using machine learning’. Further screening involved screening of the abstracts of the articles, evaluation of the titles, keywords, and publication year of the articles and full-text screening.

### Selection criteria

Selection criteria established in the study include:

1. Papers must have been written and published in English
2. The title, abstract, methodology and results of the associated literature must be in line with the subject matter. The associated literature must therefore have healthcare, management, and machine learning in their titles and/or abstract and must appear simultaneously when these keywords are equally inputted.
3. Only literature published between 2011 and 2021 must be considered. In some contexts, research published within the last ten years is considered as recent.^29^ Although this limits the body of work available, research that provides the most current evidence should only be taken into consideration.
4. Literature must have been published in journals and peer-reviewed, hence, dissertations, pre-prints, systematic reviews, and other sources are disqualified.
5. Incomplete literature, and literature without abstracts would be excluded
6. Literature must not discuss the use of machine learning in disease specific management but must be focused on healthcare institutional management and how it relates with improving patient outcomes

Literature that was not related to healthcare but had similar themes were also disqualified. For example, the keyword ‘machine learning’ might appear in a research article about the strategic management of financial institutions, this sort of document was excluded. Grey literature was deliberately avoided as articles that had not been peer reviewed, reports, dissertations, infographics, and posters were not taken into consideration.

### Data abstraction

A standardized form for data collection was developed using a comma-separated value file, and the extracted data was reviewed by an independent researcher who was knowledgeable about the subject matter. Author affiliations, publications and publishers were used as a proxy for the interest groups. The publications were classified into health management, computer science, and general, and the research questions were used as parameters for abstraction. The distribution of articles by year of publication and country of research was also noted. It was ensured that there were no duplicates, and the selection criteria were strictly adhered to. Extracted articles were arranged as data frames and properly sorted using the pandas library in python and visualized using the seaborn library that is based on matplotlib. The data elements extracted include Title, Author, Year, Publisher, Country, Type of article, applications of machine learning stated, algorithms that are in use, existing limitations of these algorithms and/or other machine learning interventions, and benefits of machine learning in the management of healthcare systems.

***Sorting out articles.*** Articles were sorted using the pandas library in python, they were categorized to reveal article information and responses to research questions.

### Article abstraction

import pandas as pd

import numpy as np

import seaborn as sea

#Sorting out Authors to title, Journals, and Year

a [‘Author Information’] = a[‘AUTHOR’] + a[‘TITLE’] + a[‘JOURNAL’] + a [‘YEAR’].astype(str)

print (a [‘Author Information’])

#Table

a [‘Author Information’].to_csv (‘Author Information use.csv’, *index *= False)

a = pd.read_csv(‘Article_data.csv’)

a.head(5)

a.describe()

#Sorting out Articles to the questions that they answered

a [‘Answers to RQ1 and RQ2’] = a [‘ARTICLE IDENTIFIERS’] + a [‘APPLICATIONS OF MACHINE LEARNING’] + a[‘ALGORITHMS IN USE’]

print (a [‘Answers to RQ1 and RQ2’])

a [‘Answers to RQ3 and RQ4’] = a [‘ARTICLE IDENTIFIERS’] + a [‘Existing limitations of these algorithms and/or other machine learning interventions’] + a [‘Benefits of machine learning in the management of healthcare systems’]

print (a [‘Answers to RQ3 and RQ4’])

### Risk of bias assessment

There are several tools used in the assessment of bias risk in research and the CASP checklist for systematic reviews which is an appraisal tool was used for this purpose. The checklist was used to assess the validity of the study, the results, and its overall beneficence. Apart from the corresponding author, two independent reviewers assessed the study.

## Results

From the four databases that were searched, Springer link provided the most results with an average of 7709 articles using the search strings. However, after the second screening (Title, keyword, publication year, and abstract screening) only twenty articles were found to be relevant, and finally five articles were reviewed after full-text screening. Screening processes involved selection of articles within the scope of this discourse with articles meeting inclusion criteria on a broad range, further screening involved ensuring that each paper could answer research questions, and that they were specifically highlighting solutions for the management of healthcare systems. No article was selected from the SCOPUS database, because although there was a plethora of results, none fit into the inclusion criteria of being specific with healthcare management.

 Table 1

**Table 1.** Results from search strings according to databases.

Database	Search string: Healthcare management and machine learning	Search string: Machine learning, big data in healthcare systems	Search string: Management of healthcare systems using machine learning	Total number of articles excluding duplicates	Number of qualified articles	Number of relevant articles	Number of articles reviewed
SCOPUS	1141	634	807	1141	115	9	0
IEEE Xplore	358	249	199	78	70	10	0
Springer Link	8901	5721	8507	8800	1104	20	5
Science Direct	107 (92 were articles in journals)	50 (34 were articles in journals)	58 (47 were articles in journals)	80	31	12	1

In line with the inclusion criteria, 51 articles were found to be relevant from the four databases. They were screened further to check for duplicates, relevance to the subject matter, and presence of necessary keywords. After the sorting process, 6 articles were abstracted and data elements were extracted and exported via a csv file to spyder, that served as the IDE through which the data were analysed using pandas and seaborn.

 Figure 4

**Figure 4.**
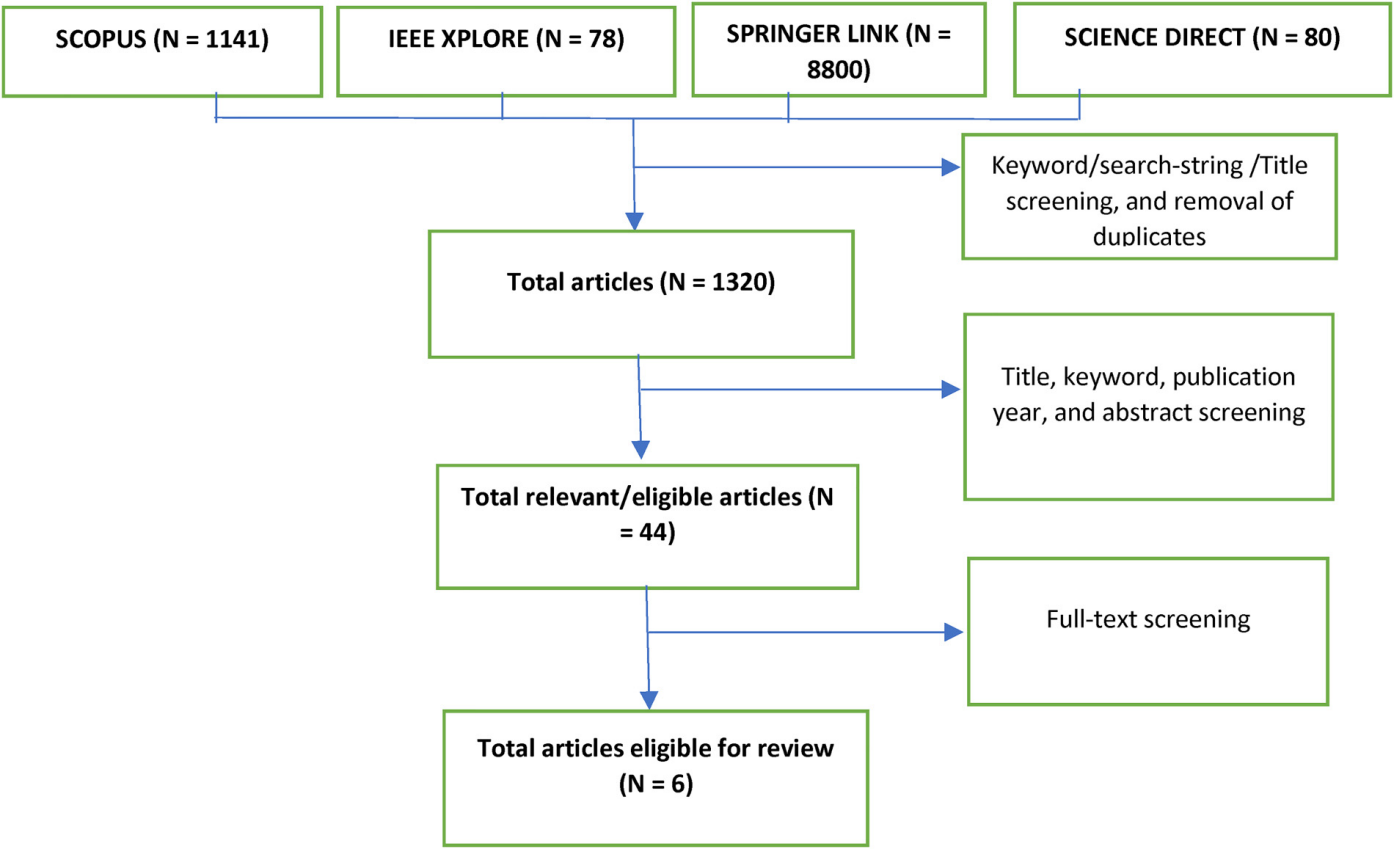
PRISMA flowchart of the process of article selection.

Upon using the selected search strings, an average of 1141 articles were discovered from the SCOPUS database, 78 articles from IEEE Xplore, 8800 articles from SpringerLink and 80 articles from ScienceDirect to broadly fit the inclusion criteria. Further screening which involved the removal of duplicates, keyword analysis, and title screening narrowed qualified articles to a total number of 1320. The titles and keywords were reviewed one more time, publication year was assessed, abstracts were fully read, and other inclusion criteria were paid attention to. However, after this stage of screening there were only 44 relevant articles and finally, after full-text screening only 6 articles were eligible for review.

 Figure 5

**Figure 5.**
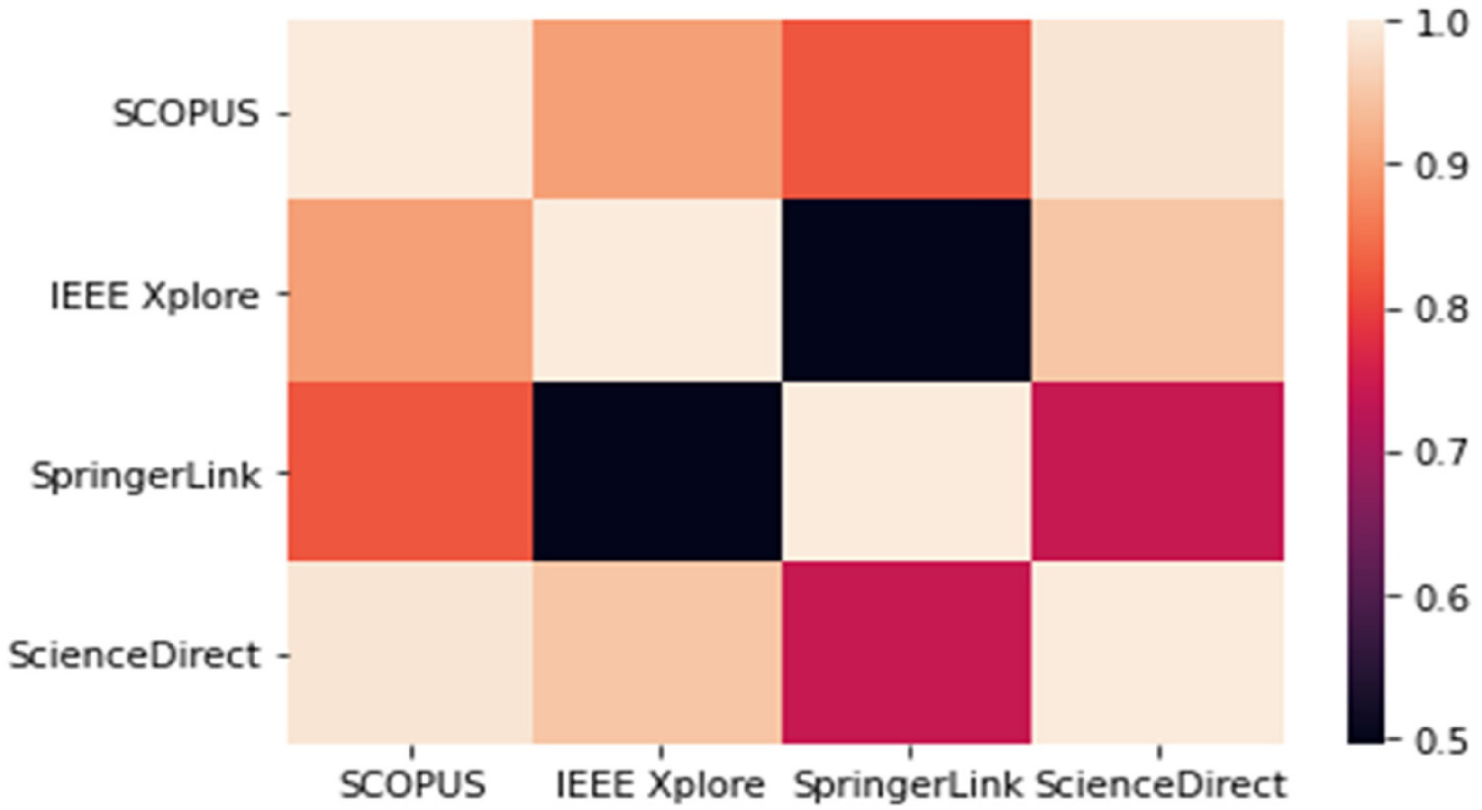
Heatmap highlighting the number of resources on each database. Source: owner.

 Table 2

**Table 2.** Article information.

*ARTICLE IDENTIFIERS*	*TITLE*	*AUTHOR*	*YEAR*
*A1*	Understanding health management and safety decisions using signal processing and machine learning	Lisa Aufegger ^30^	2019
*A2*	Big data in healthcare: management, analysis and future prospects	Sabyasachi Dash ^31^	2019
*A3*	Knowledge based dynamic cluster model for healthcare management using a convolutional neural network	Kyungyong Chung ^32^	2020
*A4*	Using machine learning classifiers to assist healthcare-related decisions: classification of electronic patient records	Juliana T. Pollettini ^33^	2012
*A5*	The basics of data, big data, and machine learning in clinical practice	David Soriano-Valdez ^34^	2021
*B1*	Impact of machine learning on management, healthcare, and agriculture	Harikumar Pallathadka ^35^	2021

### Abstracted articles

***A1 - Understanding health management and safety decisions using signal processing and machine learning.*** Research conducted by Lisa Aufegger et al.^34^ was focused on trying to understand the influence of machine learning in health management, particularly in teamwork and associated behaviours. Interactions and communication between groups were examined using a video and audio recorded role-play exercise. The healthcare professionals were classified into clinical and non-clinical groups, and thereafter organised into twenty-eight^28^ teams. A fictional healthcare system was the setting used to conduct the video/audio recorded role play exercise, this fictional environment contained problems, opportunities and issues all related to patient safety. The topic modelling approach^36^ is an unsupervised machine learning technique based on a probabilistic generative model that scans data e.g., documents automatically, detects phrase and word patterns and thereafter clusters words and similar expressions. It can be utilized in several fields one of which is text mining and tracking of epidemics.^37,38^ This approach highlights the possibility of machine learning tools being implemented in a variety of ways within a healthcare infrastructure. In this research, this modelling approach was used to analyse conversations between small groups after they had been appropriately extracted and coded. The processing of transcripts and observational data was achieved by their importation into MATLAB 2017b, and the subsequent recurrent quantification analysis based on topic modelling.^39^

Results from this study revealed that interactions among the groups were characterised by medium levels of predictability, low stability and medium to high complexity. Perceived social support was positively related to structural stability, while it was negatively related to the expectedness of the interactions sequence. Other research has highlighted the importance of understanding the perceptions and behaviour of healthcare staff^40^ as they could improve health outcomes,^41^ facilitate the adoption of technology,^42^ or the design of planning processes.^43^ There were limits within this research, as the use of self-reported data instead of expert opinion on the quality of the results might affect confirmation of usefulness and there might be reduced external validity due to the simulation of the scenario. The quality of data used in any machine learning algorithm would affect the quality of the results, it is important that data is validated^44^ and cleaned before being transformed.^45^

***A2 - Big data in healthcare: management, analysis, and future prospects*.** The research article by Sabyasachi Dash et al.^35^ paid attention to critically explaining the rudiments of big data and how healthcare is a big data repository. Much attention was paid to the advent of Electronic Health Records by highlighting advantages and possible disadvantages, all of which were related to the framework of big data analytics. Healthcare systems are gradually approaching standardized methods of personalized or precision healthcare.^46^ This article elaborates on the consolidation between generated data through multi-omics profiles, data integration and the analysis of these data in the form of electronic health records, clinical information, and patient health information, all of which are geared towards improving precision healthcare. A pivotal desideratum in the delivery of quality healthcare involves the utilization of data analytics and basic machine learning algorithms, despite the variability and complexity of healthcare operations.^47,48^

 Table 3

**Table 3.** Responses to research question 1 (RQ1) and research question 2 (RQ2).

*ARTICLE IDENTIFIERS*	*APPLICATIONS OF MACHINE LEARNING(RQ1)*	*ALGORITHMS IN USE (RQ2)*
*A1*	Signal processing and machine learning techniques were used to understand teamwork and behaviour which are related to patient safety and healthcare management.	Recurrence quantification analysis. Interactions were categorized using bales. Interaction process analysis (ipa) model, videos were coded using mangold interact software, Audio recorded sessions were transcribed and imported into MATLAB for pre-processing, and then generalised estimating equations were used for data analysis. Machine Learning: Topic Modelling approach
*A2*	The framework of Big Data Analytics and basic machine learning algorithms was used to inform the creation of Electronic Health Records	No specific algorithm mentioned, however summarized ideas are provided
*A3*	A knowledge-based dynamic cluster model serves as the foundation for the use of convolutional neural networks in healthcare management	Dynamic cluster model using a convolutional neural network
*A4*	The creation of a machine-learning software architecture-based framework that was divided into three layers and supports the automatic recommendation of patient healthcare needs based on the classification and analysis of patient information.	The second layer which is the classification layer is made up of different classification modules: K-nearest-neighbour, Relevance feedback, Artificial Neural Network, Decision Tree, and an ensemble classifier.
*A5*	Several applications were discussed under each algorithm type	Supervised, unsupervised and reinforcement learning were reviewed
*B1*	Creation of chatbots, handling patient data, medical image detection, ai assisted surgery, disease prediction, multi disease prediction	No specific algorithm mentioned, however summarized ideas are provided

***A3- Knowledge based dynamic cluster model for healthcare management using a convolutional neural network.*** Kyungyong Chung et al.^36^ were focused on utilizing a convolutional neural network for healthcare management based on a dynamic cluster model that is knowledge based. They understood how healthcare management covered diverse aspects such as health promotion, disease prevention, personalized healthcare, and overall organization, all of which are centred on the patient. Combining IT with medical services intentionally results in improved healthcare management and customized services.^49,50^ Convolutional neural networks facilitate collaborative filtering by the utilization of knowledge-based context data and structured static data (both which act as the knowledge base for services related to healthcare management), which could potentially improve healthcare management. This process conducts predictions in the healthcare settings in line with preferences of similar information within a user cluster.^51^ The dynamic cluster model is founded on knowledge based static modelling. They reported that this is preferred because of the accuracy of prediction values that arises due to similarity of information between users.^52,53^ Accuracy is therefore improved by the dynamic clustering users that have high levels of similarity.

***A4 - Using machine learning classifiers to assist healthcare-related decisions: classification of electronic patient records*.** The Brazilian healthcare system was not left out of the context when the implications of machine learning in healthcare management were being discussed by Juliana T. Pollettini et al..^37^ The research article started by providing a thorough literature review on the Brazilian healthcare network. The Sistema Único de Saúde (SUS) or public sector is controlled by the state and organized into a complex network of services. Healthcare services are essentially divided into three according to their levels of complexity. A study conducted by an Interdisciplinary Research and Teaching Group in Brazil led to the proposition of an efficient measurement for identifying healthcare needs in primary healthcare centres called the Surveillance Level (SL). The SL could be used to identify risk factors associated with patients and even recommend procedures for paediatrics. Ultimately, this research article was focused on using machine learning classifiers to recommend surveillance level scores for health records of patients, which would assist in the making of decisions as it relates to healthcare management.

A machine learning architecture-based framework which analysed and classified patient information to recommend surveillance levels was presented. This framework was classified into three layers which are the presentation, storage, and classification layers. The presentation layer supported several functionalities such as mapping projections of geo-referenced surveillance levels, surveillance level updates, recommendation of surveillance levels to healthcare practitioners as a second opinion, software setups and the visual representation of relevant information generated by the machine learning classifiers. The classification layer on the other hand is responsible for assigning surveillance levels for the patient after their information has been entered. There are two modules which make up the classification layer and each corresponds to the classification retrieval methods that process relevant information and assigns the surveillance levels automatically, courtesy machine learning. The researchers of this project supported automatic assignment of SL's, via text processing which could ultimately enhance the performance of classification. Although the research article was part of a larger project called VLIS, this research was focused on automatic SL's. This system received two new modules which were an ensemble classifier and decision tree module, compared to the first version that applied artificial neural networks and K-nearest neighbour,^54^ the second version which had a relevance feedback module^55^ and the third which basically included a linguistic module.

The machine learning architecture discussed in the research material was validated using digital data (written in Portuguese) obtained from 100 medical records of patients who presented with symptoms of a disease or who had prophylactic healthcare appointments in the year 2007. By using pattern-based classification of electronic health records, the automatic recommendation of surveillance level scores was studied. The research paper elaborated in detail how SL recommendations could be achieved using machine learning classifiers such as K-nearest Neighbour, Decision Trees, Ensemble of Classifiers (Vote), Relevance feedback, a thesaurus, and Artificial Neural Networks. Results revealed that the best classifiers were the ensemble of classifiers (Vote) because they were able to accurately classify 87.81% of patients and recommend therapeutic/medical procedures and routines. They highlighted their intention to broaden the study by utilizing larger datasets and other methods such as semantic networks. This article appears to be highly relevant as their results may be used by healthcare managers and governmental institutions to improve healthcare services.

***A5 - The basics of data, big data, and machine learning in clinical practice.*** In analysing the role of machine learning and big data in clinical practice, David Soriano-Valdez et al.^38^ discussed how big data and machine learning algorithms could be used to analyse data that would eventually benefit clinical practice, particularly rheumatology. Projects related to data, understanding the classical 7 V's (volume, veracity, validity, velocity, value, variability, and variety) of big data and Interdisciplinary conversations are being utilized to improve clinical practice. They emphasized the importance of defining units of observation, data points and units of analysis when creating a research protocol that would make use of big data and machine learning algorithms. The pros and cons of several machine learning techniques e.g., clustering,^56^ classification^57^ and regression^58^ which might be useful in the analysis of big data generated from healthcare systems were discussed, with attention being paid to reinforcement learning methods in clinical studies and its usefulness in personalized healthcare. Transdisciplinary collaborations are therefore encouraged for the successful handling of data using relevant machine learning tools to improve clinical practice in its entirety. Similarly, research conducted by^59^ Sri Venkat Gunturi et al.(94) summarizes the role of data science in the advancement of healthcare management. The conclusive summary being that the analysis of data generated by healthcare systems could aid in the making of strategic decisions that would benefit them. Healthcare quality could therefore be boosted because a comprehensive view of clinicians, patients and other stakeholders are taken into consideration before decisions are made, when these analytical tools are being utilized.

 Table 4

**Table 4.** Responses to research question^3^ and research question.^4^

*ARTICLE IDENTIFIERS*	*EXISTING LIMITATIONS OF THESE ALGORITHMS AND/OR OTHER MACHINE LEARNING INTERVENTIONS (RQ3)*	*BENEFITS OF MACHINE LEARNING IN THE MANAGEMENT OF HEALTHCARE SYSTEMS (RQ4)*
*A1*	1. The use of self-reported data 2. The use of a simulated scenario that might reduce external validity	Research revealed that: Stable interactions amongst teams regardless of how complex they are is directly proportional to an increased perceived social support. Researchers should be motivated to conduct healthy validations, where important themes from the technique of machine learning used are benchmarked against a range of ongoing established practices in health management
*A2*	1. Handling large volume of data 2. The implementation of high-end protocols, computing tools, and hardware in the clinical setting. 3. Heterogeneity of data 4. Interoperability 4. Data Sharing	The analysis of data provided by healthcare institutions can provide further insights in terms of medical, technical, procedural, and other types of improvements in healthcare.
*A3*	Setting a smaller learning rate parameter value while using the CNN algorithm in deep learning would improve accuracy but slow the speed of learning down.	Greater performance improvement was observed, and the framework established through the study can be used to configure systems in the future.
*A4*	Results, both theoretical and empirical, reveal that there is not a single algorithm that would achieve the best performance in every domain uniformly.	Several studies have provided metrics needed to rank patients, however, neither have classified patients using an automatic system nor applied machine learning to assist this activity. This offers the benefit of using classifiers of machine learning to rank patients in line with healthcare measures. Results from the research can be used by health and governmental institutions to improve health services or even alert relevant authorities about the possibility of an epidemic.
*A5*	Complex, hence requires interdisciplinary approaches	Personalized healthcare and decision-making tools
*B1*	Limitations are discussed under the types of algorithms being used	Could improve healthcare systems e.g., Disease Prediction, Multi disease prediction, AI Assisted Surgery and Personalized Medicine

***B1- Impact of machine learning on management, healthcare, and agriculture.*** Harikumar Pallathadka et al.^39^ discussed the impact machine learning has on the management of healthcare systems by elaborating on two different types of models (generative e.g., Naïve Bayes and discriminative e.g., Radial Bias) and establishing their relationship with healthcare system management. Several benefits of machine learning and artificial intelligence were highlighted, and they include the prediction of diseases, personalized medicine, and medical image detection. The risks shared in this empirical research article remain similar with previously mentioned risks; being notably, privacy and security issues. Within a secure healthcare framework, Prableen Kaur et al.(95) discussed how the data generated by healthcare systems, which could be analysed by machine learning/artificial intelligence, could be managed by using a secure healthcare information system. Several machine-learning based health information systems have been designed, and even a study proposed by Arwinder Dhillona et al.(97) revealed how they could be used to predict addiction to alcohol. The framework proposed by Ahmed E. Youssef(101) consists of five major components which are the cloud environment, the electronic health record, the security layer, big data analytics and the information delivery layer. Several risks associated with security and privacy are mitigated, and ultimately if this is overlapped with the framework proposed by Prableen Kaur et al.(95), the machine learning based layer would become more tangible. This layer has 5 sub-modules called drug discovery, early diagnosis, data analytics, and visualization, alongside outbreak forecasting, and uses different machine learning algorithms such as Naïve Bayes, genetic algorithms, decision trees and support vector machines etc. These machine learning techniques could be combined with other techniques to yield great results, while also ensuring data security and privacy.

## Discussion

Management has been defined over the years in different ways by various researchers, some of these definitions include referring to management as a process that involves activities occurring within organizations with the aim of accomplishing pre-defined objectives,^60^ the ability to transform resources into utility^61^ or even the process of being responsible for and supporting work performance.^62^ Regardless of the definition, based on Henri Fayol's perspective, the core functions of a manager are planning, organizing, staffing, controlling, directing and decision making,^63^ and there are a plethora of managerial positions. In the healthcare setting, some of these positions would include a chief medical director, clinical nurse manager, pharmacy manager, director of medical records etc.^64^ Bob Nelson^65^ extended the functions of a manager to include energizing, supporting, empowering, and communicating with employees; apparently, all of these would contribute to having a sustainable management system.^65^

It has been stated that effective management of a healthcare institution can only be achieved by the proper conduction of all managerial functions at three levels which are: self, team, and the organization**,**^64^ and these would inevitably lead to an improvement of patient outcomes.^66^ Ethics, human resource management, healthcare financing and budgeting, strategic planning and marketing are essential parts of a healthcare institution that should not be ignored.^67^ A conglomerate of proper functioning healthcare institutions provides evidence of an optimized health system in which each building block is catered for.^4^ Health institutions e.g., hospitals, pharmacies, care homes etc. generate tonnes of data such as discharge summaries, insurance information, medical images, laboratory results, social media information, hospital admission notes, the internet of things etc., and these have become very complex to manage. Due to the colossal growth of data and the speed with which they are generated, traditional methods of processing and analysis have become obsolete and other forms of technology must be optimized.^68^ As mentioned in the introduction, analysis of this data could provide information that would be useful in making better decisions, improving staff recruitment, human resource, and supply chain management, as well as proper integration of existing health management frameworks with new innovative technologies. Therefore, it is important to understand that achieving present and future health needs can be achieved by the development of fields that would facilitate the optimization of available data.^31^ The onset of cloud computing has aided the management of big data in the aspect of storage, networking, and security over the internet, but the analysis of the data itself can only be achieved through data analytics and data science. Data science is of primary interest because of its ability to predict future outcomes and proffer solutions, and this would be of uttermost importance to managers of health facilities.

There are a good number of articles describing the importance of data science in health,^69–72^ but it was noticed that there was little on the actual management of the health facilities. Also, most recommendations were disease specific or just focused on basic electronic health records– almost disregarding the link between an optimum managerial framework, healthy working environment, and patient outcomes. There are different aspects of data science such as cloud computing, data engineering, database management and infrastructure, and machine learning, and they all work together to ensure that data is optimized efficiently. Machine learning is a growing field that permits computers to learn from available data to make predictions, results can be visualized, and algorithms can be used to design different applications that would influence managerial processes with improved patient outcomes being the desired result. Data could be used to predict pandemics,^59,73,74^ and machine learning tools could be used to generate solutions for pandemic management, marketing of pharmaceutical products, or the dissemination of vaccines. Propaganda grows in the absence of clarity, and preventable mistakes would be inevitable in absence of a feasible framework– these are problems that proper analysis of health driven data using machine learning could mitigate.^75,76^

The articles that were eventually reviewed after the critical selection process were enlightening. The research conducted by Lisa Aufegger et al.^30^ involved the use of machine learning to understand the behaviour of both clinical and non-clinical staff as it relates to healthcare management, and although limitations such as resource- and time- intensive data processing exist, the objectives of the study were still accomplished. The analysis of qualitative data using machine learning (topic modelling approach) to identify emerging themes was daring because there is limited information on the use of this technique in understanding conversations related to patient safety and healthcare management. Studies have demonstrated that this approach is satisfactory for analysing data from open-ended responses and blogs.^77,78^ In fact, evaluation of the results of a workforce survey conducted by Deloitte and analysed by a similar machine learning algorithm pinpointed that although the algorithm was faster at screening data, the quality of its output was inferior.^79^ Hence, the success of this study in comparing its results with previous studies utilizing methods unrelated to machine learning and observing high levels of similarity, sheds more light on the possible utilization of machine learning in healthcare management and patient safety. This creates room for further research and its possible adoption as a tool for healthcare managers.

The research conducted in Brazil by Juliana T. et al.^33^ on the use of machine learning classifiers in assisting the decision-making processes in healthcare institutions was equally apt because a machine learning algorithm was actively utilised in the categorisation of electronic health records. Prior to their research being published, there were no studies that involved the combination of machine learning algorithms and surveillance systems, and they were meticulous enough to identify specialized vocabulary used by their participants. It was proposed that results from this system with an adequate framework would aid healthcare managers in conducting their duties appropriately and could even be used to predict an epidemic.

### Limitations

There are several limitations of this study, the first being the exclusion of grey literature which contained articles that had not been peer-reviewed, conference papers, reports, and dissertations, some of which could have potentially added to the robustness of this scoping review. Although the databases selected possess journals that are predominantly focused on big data, machine learning and health management, there might be limitations due to the restriction to only certain types of articles made available in those journals. Regardless, using multiple databases might complicate search strategies and syntaxes. It is therefore suggested that an extensive review that would cover other databases is conducted.

## Conclusion

In this study, a comprehensive review of the use of machine learning in the management of healthcare systems are provided. Research reveals that machine learning has a cornucopia of applications within the healthcare infrastructure such as medical imaging, personalized medicine, and AI-assisted surgeries, unfortunately it appears that little attention is paid to the utilization and optimization of machine learning in the management of health systems. This is because there are limited studies and research concerned with the use of machine learning in improving the managerial aspects of health institutions. Therefore, it would be interesting to witness machine learning not just being useful in applications such as personalized medicine for the benefit of the patient,^80^ but as the basis for the design of a healthcare management framework and as a panacea to improving baseline approaches to patient-staff management within a healthcare setting. For example, using an advanced topic modelling approach (a machine learning algorithm), the perceptions of healthcare staff could be understood to improve their adoption to latest technology or the acceptance/implementation of structural frameworks. Also, machine learning classifiers could be used to recommend surveillance systems which would inevitably assist in the making of healthcare related management decisions. The influence of machine learning in other aspects of managing healthcare institutions such as risk management, staffing, supply chain management, change management and enhanced patient engagement with evolving innovations, should be adequately explored. While this would expand the possibilities of machine learning as a growing field in data science, it would improve the quality of healthcare institutions which would inevitably affect patient outcomes.
